# Development of a Novel Prognostic Model for Lung Adenocarcinoma Utilizing Pyroptosis-Associated LncRNAs

**DOI:** 10.1155/ancp/4488139

**Published:** 2025-01-13

**Authors:** Hong-Yan Bai, Tian-Tian Li, Li-Na Sun, Jing-Hong Zhang, Xiu-He Kang, Yi-Qing Qu

**Affiliations:** Department of Pulmonary and Critical Care Medicine, Qilu Hospital, Cheeloo College of Medicine, Shandong Key Laboratory of Infectious Respiratory Diseases, Shandong University, Jinan, China

**Keywords:** bioinformatics, immune, lung adenocarcinoma, prognosis, pyroptosis

## Abstract

Lung cancer is a highly prevalent and fatal cancer that seriously threatens the safety of people in various regions around the world. Difficulty in early diagnosis and strong drug resistance have always been difficulties in the treatment of lung cancer, so the prognosis of lung cancer has always been the focus of scientific researchers. This study used genotype-tissue expression (GTEx) and the cancer genome atlas (TCGA) databases to obtain 477 lung adenocarcinoma (LUAD) and 347 healthy individuals' samples as research subjects and divided LUAD patients into low-risk and high-risk groups based on prognostic risk scores. Differentially expressed gene (DEG) analysis was performed on 25 pyroptosis-related genes obtained from GeneCards and MSigDB databases in cancer tissues of LUAD patients and noncancerous tissues of healthy individuals, and seven genes were significantly different in cancer tissues and noncancerous tissues among them. Coexpression analysis and differential expression analysis of these genes and long noncoding RNAs (lncRNAs) found that three lncRNAs (AC012615.1, AC099850.3, and AO0001453.2) had significant differences in expression between cancer tissues and noncancerous tissues. We used Cox regression and the least absolute shrinkage sum selection operator (LASSO) regression to construct a prognostic model for LUAD patients with these three pyroptosis-related lncRNAs (pRLs) and analyzed the prognostic value of the pRLs model by the Likaplan–Meier curve and Cox regression. The results show that the risk prediction model has good prediction ability. In addition, we also studied the differences in tumor mutation burden (TMB), tumor immune dysfunction and rejection (TIDE), and immune microenvironment with pRLs risk scores in low-risk and high-risk groups. This study successfully established a LUAD prognostic model based on pRLs, which provides new insights into lncRNA-based LUAD diagnosis and treatment strategies.

## 1. Introduction

Lung cancer is the most common cancer in the world, with the highest morbidity and mortality rates among all cancers [1, 2]. Lung cancer patients can be divided into two major categories based on their pathological and clinical characteristics, namely small cell lung cancer (SCLC) [3] and nonsmall cell lung cancer (NSCLC) [4]. NSCLC is the most common type of lung cancer, accounting for 85% of lung cancer patients. NSCLC can be further divided into lung adenocarcinoma (LUAD) [5], lung squamous cell carcinoma [6], and large cell neuroendocrine carcinoma [7], and LUAD is the most common subtype of NSCLC. Many treatments can be used to treat LUAD, such as surgical resection [8], chemotherapy [9], radiotherapy [10], molecular targeted therapy, and immunotherapy [11]. However, early diagnosis of LUAD is difficult, and its high drug resistance and easy metastasis bring many difficulties to the treatment of lung cancer.

Pyroptosis is a form of inflammatory programed cell death [12], which is characterized by continued cell swelling until the cell membrane ruptures, subsequently releasing intracellular contents and triggering an inflammatory response. Many studies have shown that pyroptosis also plays an important role in the occurrence and development of various diseases, such as infectious diseases [12], immune diseases [13], and cancer [14]. In tumors, pyroptosis can eliminate tumor cells and inhibit tumor growth and metastasis [15]. Pyroptosis stimulates antitumor immune activity in the body by releasing pro-inflammatory cytokines and immunogenic substances that can activate and attract immune cells into the tumor site [15]. A study on radiotherapy for cancer found that radiotherapy can induce cancer cell pyroptosis to enhance antitumor immune effects [16]. A study improves the efficiency of pyroptosis in cancer treatment by designing bioorthogonal pyroptosis nanoregulators to promote pyroptosis and disrupt the self-protection mechanism of cancer cells [17]. However, some tumor cells can escape the induction of pyroptosis and promote tumor development through different pathways, such as regulating the inflammasome signaling pathway and regulating the expression of Gasdermin D (GSDMD) protein [18]. Moreover, the cytokines IL-1 and IL-18 released during pyroptosis can promote tumor infiltration and increase the possibility of tumor development and metastasis [19].

Long noncoding RNA (lncRNA) is a type of RNA molecule that is more than 200 nucleotides in length and cannot encode proteins [20]. Although lncRNA cannot encode proteins, they can perform various biological functions, such as regulating gene expression, chromatin modification, transcriptional regulation, and posttranscriptional regulation [21–23]. In recent years, research on lncRNA has shown that they play a key role in the occurrence and development of various diseases, especially their role in cancer has attracted much attention [24]. Many lncRNAs have been found to be involved in the regulation of malignant biological behaviors such as cancer cell proliferation, invasion and metastasis, and drug resistance [25, 26]. Moreover, some lncRNAs also show regulatory effects on the tumor microenvironment (TME), affecting tumor immune escape, angiogenesis, matrix remodeling, and other processes [27, 28]. Metastasis-associated LUAD transcript 1 (MALAT1) promotes the transformation of tumor cells to an invasive phenotype and drives distant metastasis by regulating a complex network of transcription factors, signaling pathways, and genes associated with epithelial-mesenchymal transition (EMT) [29]. Due to the unique functions of lncRNAs, lncRNAs have been widely used in cancer prognostic models, such as survival prediction models [30], treatment response prediction models [31], and recurrence risk assessment models [32].

This study used LUAD patients and healthy individuals from the genotype-tissue expression (GTEx) and the cancer genome atlas (TCGA) databases as the research subjects and obtained 25 pyroptosis-related genes from the GeneCards and MSigDB databases. Differential genes and lncRNAs were screened out for coexpression analysis to identify pyroptosis-related lncRNAs (pRLs) differentially expressed in LUAD patients and healthy individuals. We used these three pRLs to form a prognostic model for LUAD patients and evaluated the accuracy of this model. This study screened three lncRNA features related to pyroptosis as prognostic predictors, which is of great significance for personalized treatment strategies for LUAD patients and for improving the accuracy of the clinical prognosis of LUAD.

## 2. Materials and Methods

### 2.1. Data Sources

The data sources used in this study are gGTEx [33] and TCGA databases [34], and we downloaded relevant patient data from UCSC Xena (https://xena.ucsc.edu/) [35]. The subjects of this study include 477 LUAD samples from TCGA's LUAD data set, 59 noncancer control samples from the TCGA database, and 288 noncancer control samples from healthy individuals in the GTEx data, and we used Perl 5.30.0 (https://www.perl.org/) to preprocess the raw RNA-seq transcriptome data we obtained. During the quality check, samples with more than 10% missing values and less than 10 million sequencing reads in RNA-seq data were removed to avoid the impact of incomplete data sets on the analysis. In addition, genes that were expressed in at least 80% of the samples should be retained after filtering, and genes with an average TPM of less than 1 should be removed. The transcripts per million (TPM) normalization method normalizes the expression levels between different samples to ensure that the data can be used for subsequent analysis.

### 2.2. Screening of Pyroptosis-Related Genes

GeneCards [36] is a database of human genetic information that contains gene function, expression patterns, disease associations, and pathway information. We collected genes related to pyroptosis from the GeneCards database using the keyword “pyroptosis.” We also filtered these genes based on correlation scores and retained genes with correlation scores higher than 7 for a total of nine genes. In addition, we obtained 20 genes related to thermal decomposition from the molecular signature database (MSigDB) (http://www.gsea-msigdb.org/gsea/msigdb/) [37]. CD-hit [38] was used to remove overlapping genes, and we finally obtained 25 unique genes related to thermal degradation. These 25 genes have been linked to cancer in many studies [39, 40], especially in LUAD [41, 42].

### 2.3. Differential Expression Analysis and Exploration of Coexpression of LncRNA

According to the 25 pyroptosis-related genes we found, this study used the R package “Limma” [43] to identify the differentially expressed genes (DEGs) of pyroptosis-related genes in lung cancer tissues of LUAD patients and noncancerous tissues of healthy groups. The standardized data were fitted to the expression value of each gene by a linear model, and the statistical test results were robustly adjusted using an empirical Bayesian method (significance threshold was set at FDR < 0.05). Moreover, we conducted a coexpression network analysis on these pyroptosis-related DEGs and their target lncRNAs to identify pRLs. The coexpression network was constructed by calculating the Pearson correlation coefficient matrix between genes to screen for highly correlated gene–gene or gene-lncrNA interactions (correlation coefficient > 0.6 or *p* < 0.01). Cytoscape [44] was used to construct gene and lncRNA coexpression networks to visualize and analyze the interactions between them.

### 2.4. The Risk Model of pRLs

The Cox regression model [45] was used to analyze the correlation between clinical data of LUAD patients and pRLs gene expression results to determine the pRLs related to LUAD prognosis. For the pRLs related to LUAD prognosis, we used the least absolute shrinkage sum selection operator (LASSO) [46] analysis to establish a prognostic feature model related to pRLs in LUAD patients. The computational formula is as follows:

  $$\begin{array}{c} \text{The risk score}=\sum_{i=1}^{n}\left(\text{coefi }\times \text{ Expri}\right). \end{array}$$

Expri represents the expression value of the pRLs in the signature for patient I, and coefi represents the LASSO coefficient of the pRLs. Based on the formula, a risk score was calculated for each patient. Based on the formula, the impact of the expression of pRLs on the corresponding risk score of each LUAD patient can be calculated.

### 2.5. Identification and Verification of Pyroptosis-Related Predictive Signatures in LUAD

According to the risk model of pRLs, we calculated the risk scores of 477 LUAD patients. By calculating the median risk score of these 477 LUAD patients, 477 LUAD patients were divided into high-risk and low-risk groups based on the median risk score. Moreover, we used the area under the curve (AUC) values and receiver operating characteristic (ROC) curves to evaluate the prediction model of this study and analyzed the correlation between risk scores and clinicopathological variables to compare the clinical outcomes of low-risk patients and high-risk patients' features. In total, 477 LUAD patients were randomly divided into two groups (*n*1 = 240, *n*2 = 237) to verify the prognostic characteristics of overall survival (OS) in these cohorts.

Univariate and multivariate Cox regression analyses were used to analyze the prognostic correlation between risk scores and variables such as gender, age, and disease stage in three groups of LUAD patients (*n*1 = 240, *n*2 = 237, *n* = 477). We also constructed nomogram-based prediction models [47] (R packages RMS [48]) to predict the 1–, 3-, and 5-year OS rates of three groups of LUAD patients. A score for each independent prognostic factor is calculated based on its impact on the patient's clinical outcome, and these scores are then summed to create a total score. Calibration plots were used to evaluate the accuracy of nomograms in predicting 1-, 3-, and 5-year OS of LUAD patients [49]. Principal component analysis (PCA) was used to assess the dimensional structure of the data.

### 2.6. Functional Enrichment Analysis of the pRLs

In this study, Gene Set Enrichment Analysis (GSEA) 4.1.0 [50] was used to perform GO [51] and KEGG [52] (screening criteria: *p*-value less than 0.05 and FDR *q* value less than 0.25) enrichment analysis on genes in the high-risk group and low-risk group to determine any differences in functional enrichment between the two groups. Finally, we propose the top 5 pathways that meet the criteria of *p*-value less than 0.05 and FDR *q*-value less than 0.25.

### 2.7. TME and Immune Response Analysis

We first collected mutation data for all lung tissue samples from the TCGA database (https://portal.gdc.cancer.gov/) and used a Perl (http://www.perl.org/) script to calculate the tumor mutation load of each tumor sample (tumor mutation burden [TMB]). In order to explore the relationship between pRLs and the immune system, we used the R package reshape2 (https://github.com/cran/reshape2/) and ggpubr [53] of the estimation algorithm to perform a differential analysis of the TME and immune cell components between the high-risk and low-risk groups of LUAD patients. We also utilized the R packages ggpubr [53] and shape2 to examine differences in immune-related functions between low-risk and high-risk groups. According to the median risk score, we divided the LUAD samples into high-TMB group and low-TMB group to compare the four groups (high-TMB + high-risk score, high-TMB + low-risk score, low-TMB + high-risk score, low-TMB + low-risk score) survival rate of patients.

### 2.8. An Analysis of Drug Sensitivity in Patients Categorized as Low- and High-Risk

The R package pRRophetic (https://github.com/viparc/prophylactic) was used to evaluate the half-maximal inhibitory concentration (IC_50_) values of chemotherapy drugs in high-risk and low-risk groups to evaluate the application value of prognostic features in predicting clinical LUAD treatment and used the R packages ggplot2 [54] and ggubr to visualize the data.

### 2.9. Cell Culture

The two LUAD cell lines A-549, NCI-H1975, and human bronchial epithelial cells 16HBE were procured from Wuhan Pronosai Life Sciences Co. Ltd. (Wuhan, China). The PC-9, A-549, and 16HBE cell lines were cultured in RPMI-1640 medium supplemented with 10% fetal bovine serum (FBS). The culture of NCI-H1975 cells was performed in DMEM supplemented with 10% FBS. We kept the cells at 37°C in 5% CO_2_ and changed the medium regularly.

### 2.10. Extraction of RNA and qRT-PCR Analysis

RNA was isolated using TRIZOL (Trizol, THERMO 15596026). Following the manufacturer's protocol, we converted the total RNA samples into complementary DNA (cDNA) using the Total-Transcriptome cDNA Synthesis Kit (AG11707, Accurate Biology, China). We then performed quantitative real-time PCR (qRT-PCR) on the Bio-Rad CFX96 real-time system, using Accurate Biotech's SYBR Green Premix Pro Taq HS qPCR Kit (AG11701). In this study, GAPDH served as the reference gene. An expression level for each gene was calculated by using the 2^−ΔΔCT^ method. The primer sequences for real-time PCR are displayed in Table S1.

## 3. Results

### 3.1. Differential Expression Analysis and Coexpression Between Pyroptosis-Related Genes and LncRNA

**Figure 1:**
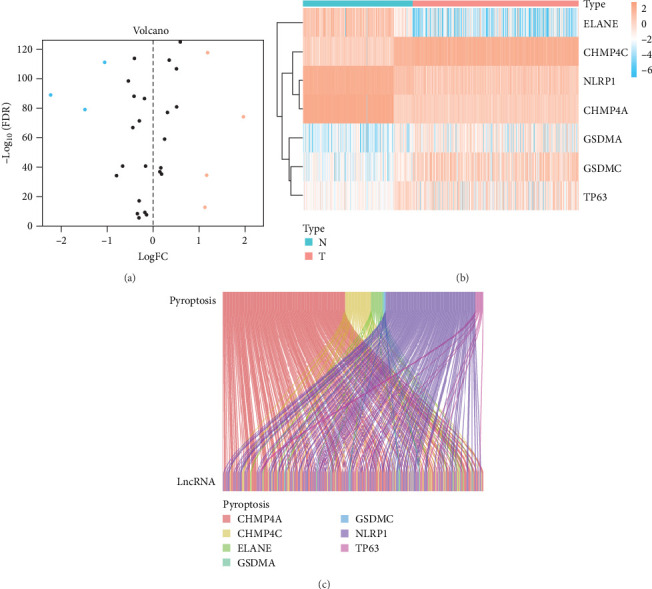
Identification of pyroptosis-related genes DEGs and coexpression analysis of between pyroptosis-related genes lncRNA. (A) Differential expression analysis of pyroptosis-related genes in noncancerous tissues of healthy individuals and cancer tissues of LUAD patients. (B) Expression profiles of pyroptosis-related DEGs in healthy individuals and LUAD patients. *Note:* N, healthy individuals; T, LUAD patients. (C) Coexpression analysis of pyroptosis-related DEGs and lncRNA. CHMP4A, charged multivesicular body protein 4A; CHMP4C, charged multivesicular body protein 4C; DEGs, differentially expressed genes; ELANE, elastase neutrophil expressed; GSDMA, gasdermin A; GSDMC, gasdermin C; LncRNA, long noncoding RNA; LUAD, lung adenocarcinoma; NLRP1, NLR family pyrin domain containing 1; TP63, tumor protein p63.

We obtained a total of 25 unique pyroptosis-related genes from GeneCards and MSigDB databases and conducted DEG analysis of these 25 genes in cancer tissues of LUAD patients and noncancerous tissues of healthy individuals (Figure 1A). The analysis results of DEGs showed that 7 of the 25 genes were differentially expressed in cancer tissues and noncancerous tissues. Among them, the expression levels of four genes were increased, and the expression levels of three genes were decreased. Further analysis of the expression levels of these seven genes in all LUAD and healthy individuals (Figure 1B), we found that charged multivesicular body protein 4C (CHMP4C), tumor protein p63 (TP63), gasdermin A (GSDMA) and gasdermin C (GSDMC) compared with the expression levels of noncancerous tissues of healthy individuals were significantly increased in the cancer tissues of LUAD patients (*p* < 0.05), while elastase neutrophil expressed (ELANE), charged multivesicular body protein 4A (CHMP4A) and NLR family pyrin domain containing 1 (NLRP1) (*p* < 0.05) were significantly decreased in the cancer tissues of LUAD patients. The coexpression relationship between 7 pyroptosis-related genes and lncRNA indicated that these genes are coexpressed with lncRNA (Figure 1C) (*p* < 0.05), especially the CHMP4C gene (*p* < 0.001). There is a strong coexpression relationship between the CHMP4A gene and lncRNA, while there is a relatively weak coexpression relationship between the GSDMC gene and lncRNA. This coexpression relationship may imply that lncRNA plays a key regulatory role in regulating the expression of pyroptosis-related genes and regulating the process of pyroptosis.

### 3.2. Developing a Prognostic Signature Based on the pRLs

**Figure 2:**
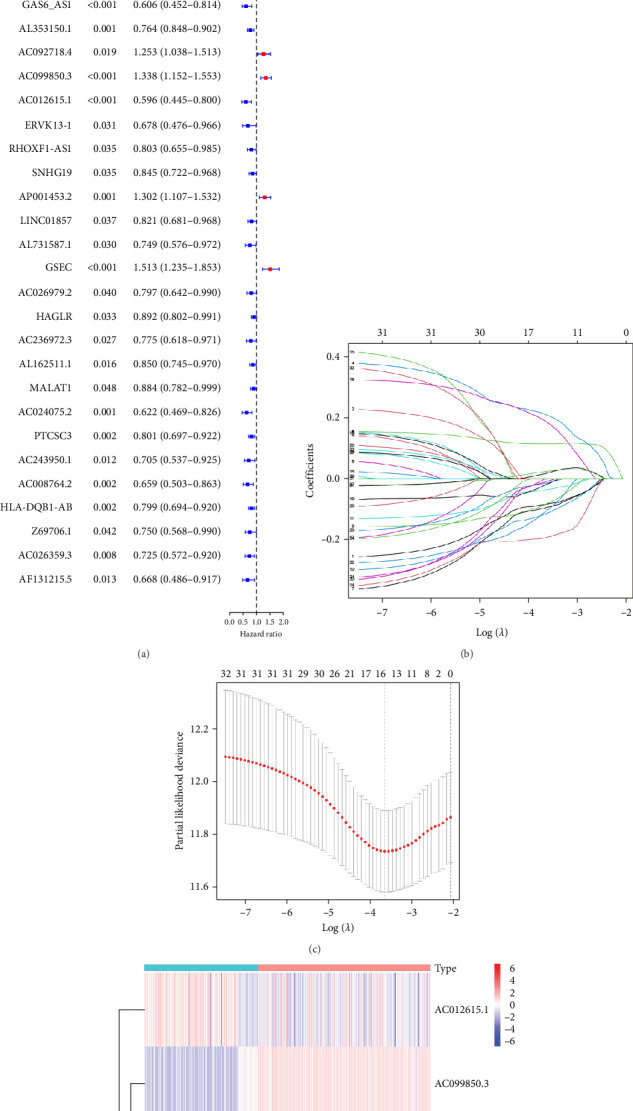
Design of a pyroptosis-associated lncRNA risk model. (A) Cox regression analysis to identify prognostic lncRNAs. (B) Cross-validation to select the optimal parameter (lambda). (C) Pyroptosis-related lncRNAs with LASSO coefficients. (D) A heatmap of gene expression related to pyroptosis. LASSO, least absolute shrinkage sum selection operator; LncRNA, long noncoding RNA.

**Figure 3:**
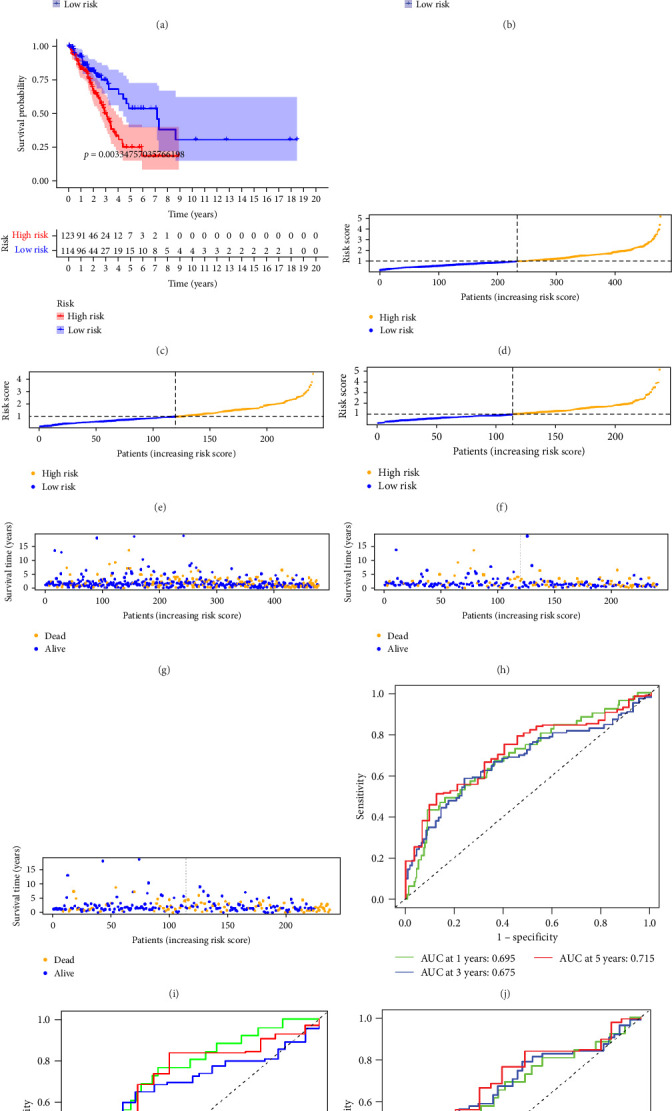
The prognostic value of the pRLs in LUAD. (A–C) The K–M curve of the predictive model in the entire set, the first internal queue and the second internal queue. (D–F) Distribution of risk scores in the entire set, the first internal queue and the second internal queue. (G–I) Distribution of survival status of patients in the entire set, the first internal queue and the second internal queue. (J–L) ROC analysis showed the AUC of the model in the entire set, the first internal queue and the second internal queue. AUC, area under the curve; LUAD, lung adenocarcinoma; ROC, receiver operating characteristic curve.

We performed univariate Cox regression analysis between the expression levels of these pRLs and the risk score of LUAD to explore the pRLs associated with LUAD (Figure 2A). The results of univariate Cox regression analysis showed that there are 34 pRLs related to LUAD, the Hazard ratio value of 6 lncRNAs, and the probability of LUAD disease exceeds 1. LASSO penalized Cox regression analysis of 34 pRLs expression levels and survival rates in LUAD patients (Figure 3B,C) and identified 16 representative pRLs. Only 3 (AC012615.1, AC099850.3, and AO0001453.2) of these 16 representative pRLs had significant differences in expression levels in cancer tissues of LUAD patients and cancer tissues of healthy individuals (Figure 3D) (*p* < 0.05). The expression levels of AC099850.3 and AO0001453.2 were higher in cancer tissues than in noncancerous tissues, while the expression levels of AC012615.1 were lower in cancerous tissues than in noncancerous tissues. This further indicates that these 16 pRLs are associated with the survival status of LUAD patients.

### 3.3. Prognosis Prediction for Patients With LUAD Using pRLs

To verify the accuracy of this model in predicting the prognostic value of LUAD, we analyzed its prognostic value in the entire cohort (*n* = 477) and two test cohorts (*n*1 = 240, *n*2 = 237) (Figure 3). In the Kaplan–Meier survival curves (Figure 3A–C) of the entire cohort (*n* = 477) and the two test cohorts (*n*1 = 240, *n*2 = 237), the low-risk group in all cohorts had better prognosis and longer life. Risk distribution results (Figure 3D–F) showed a clear association between risk score and patient outcome in the entire cohort and in both test cohorts (*p* < 0.05), and the analysis of survival time (Figure 3G–I) showed that the lower the risk score of LUAD patients in the three cohorts, the longer their survival.

We also used ROC curves to verify the reliability of the model in predicting survival rate. The AUC values of the entire cohort at 1, 3, and 5 years were 0.695, 0.675, and 0.715 (Figure 3J). The AUC values of the first internal cohort at 1 year, 3 years, and 5 years were 0.744, 0.658, and 0.738 (Figure 3K), and the AUC values of the second internal cohort at 1 year, 3 years, and 5 years were 0.649, 0.677, and 0.703 (Figure 3L). The survival rates of the entire cohort (*n* = 477) and the two test cohorts at 1, 3, and 5 years were not significantly different, indicating that this feature has certain potential characteristics in predicting the prognosis of LUAD patients.

### 3.4. Clinical Outcome for Patients With LUAD May Be Predicted by Risk Scores

**Figure 4:**
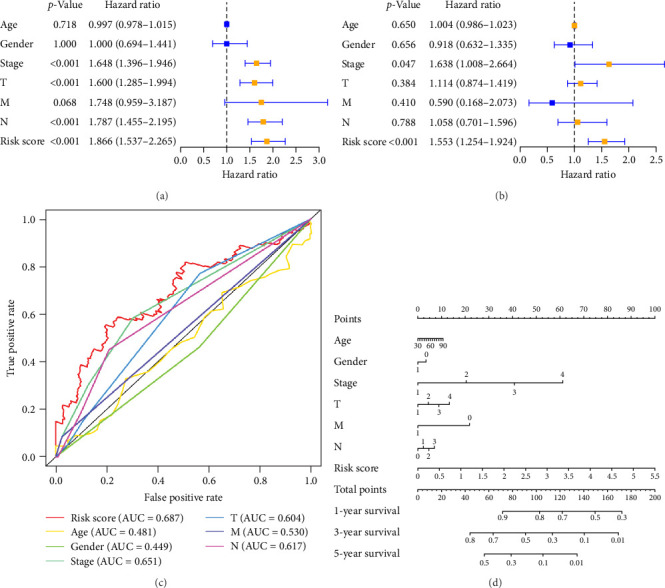
Correlation between risk score and prognosis. (A) Single-factor independent prognostic analysis of LUAD. (B) Multifactor independent prognostic analysis of LUAD. (C) ROC curve AUC for risk model score and clinical features. (D) Nomogram construction. AUC, area under the curve; LUAD, lung adenocarcinoma; M, metastasis; N, node lymph node involvement; ROC, receiver operating characteristic curve; T, tumor size and scope.

We performed univariate and multivariate Cox regression analyses on risk scores and clinical characteristics to evaluate their potential as independent prognostic factors. Univariate Cox regression analysis showed that stage, tumor size, and scope (*T*), Node lymph node involvement (*N*), and risk scores had a significant impact on prognosis (*p* < 0.001) when considering these clinicopathological characteristics (Figure 4A). The results of multivariable Cox regression analysis showed that pyroptosis-related risk score was an important independent prognostic factor (Figure 4B). Univariate and multivariate regression analysis showed that the pyroptosis-related risk score was related to the prognostic indicators of LUAD patients, and the prognostic survival status of LUAD patients was directly proportional to the risk score.

**Figure 5:**
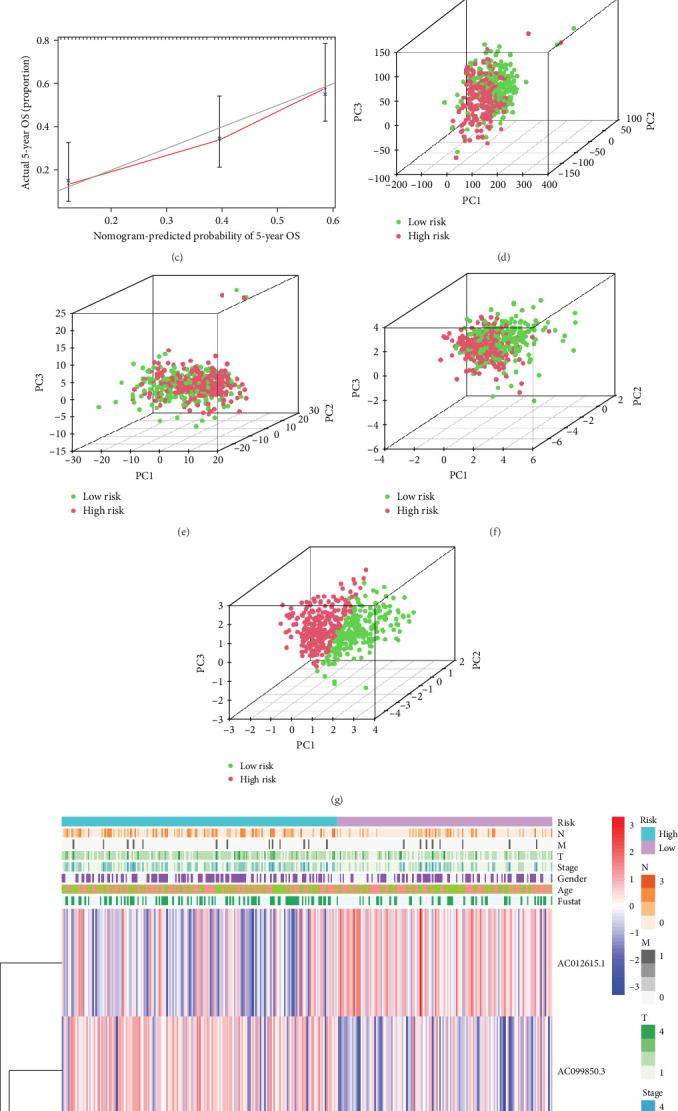
Analyzing how well LUAD predicts clinicopathological outcomes. (A–C) Calibration curve of nomogram. (D–G) PCA plots depicted a distinct distribution of high- and low-risk groups based on all genes, pyroptosis genes, pyroptosis-related lncRNAs, and pyroptosis-related lncRNAs prognostic signature. (H) Heatmap of clinicopathological features of LUAD. LncRNAs, long noncoding RNAs; LUAD, lung adenocarcinoma; OS, overall survival; PCA, principal component analysis.

To further explore the reliability of the three pRLs as prognostic factors for LUAD, we used ROC curves to analyze the survival rates of these clinicopathological features and risk scores (Figure 4C). The AUC value of the risk score is the highest, 0.687. The AUC value of gender is the lowest, 0.449. This indicates that its predictive ability as a prognostic factor for LUAD is strong, while gender performs poorly in this regard. This result is consistent with the results of single-factor and multifactor analysis, indicating that the risk score can be used as a prognostic indicator for LUAD. In addition, we constructed a nomogram to integrate the risk score and various clinicopathological features to help clinicians predict patient prognosis (Figure 5D). From the nomogram, we found that risk score has the greatest impact on patient prognosis, followed by stage, which is consistent with the results obtained from the ROC curve.

We also demonstrated the consistency of the nomogram with OS prediction at 1, 3, and 5 years by using calibration plots at 1, 3, and 5 years (Figure 5A–C), and the results showed a good consistency between them. PCA analysis was performed on all genes, pyroptosis genes, pRLs, and pRLs prognostic signatures (Figure 5D–G) of high- and low-risk groups. The PCA results showed that pRLs and pRLs prognostic signatures can be divided into two clusters, and the result that pRLs prognostic signature is divided into two clusters is more obvious than the result of pRLs. The results of this analysis highlight the superior discriminative power of the risk model, demonstrating its ability to effectively differentiate between low-risk and high-risk groups. This confirms with the findings that the identified pRLs are reliable for model construction. Comparing the expression levels of the three pRLs in this study with various clinical pathological factors (Figure 5H), we found that the expression levels of the three pRLs are related to various clinical pathological factors, which also indicates that the three pRLs may have a role in the treatment of LUAD.

### 3.5. GSEA Pathway Analysis Results of the Prognostic Model

**Figure 6:**
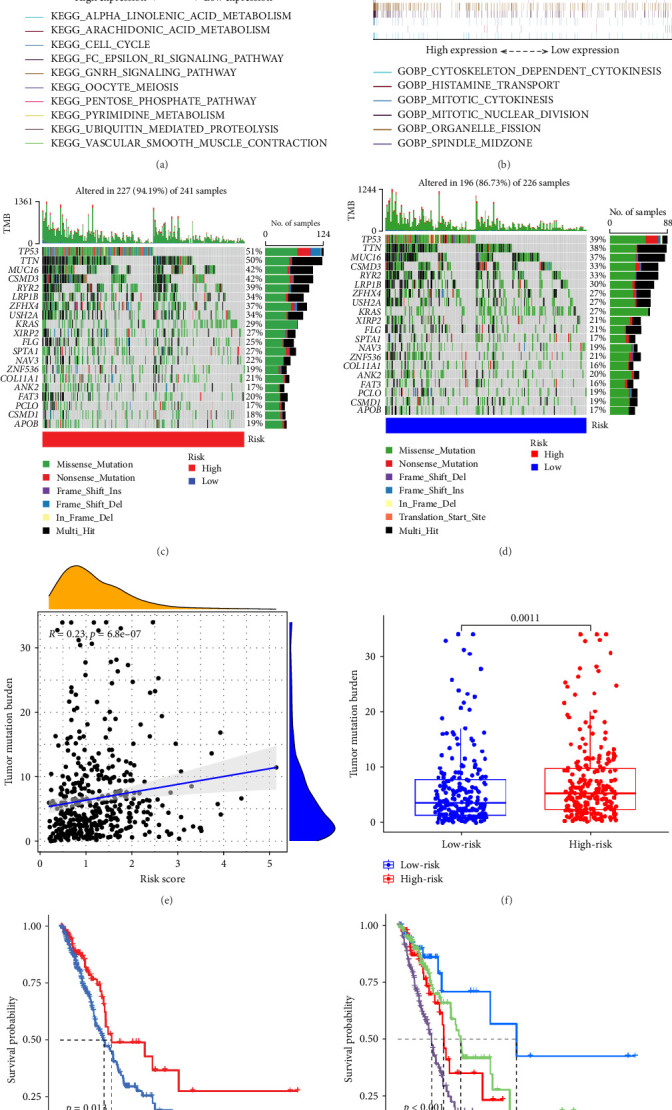
Analysis of GSEA enrichment and TMB differences and prognosis. (A) GSEA results in the KEGG analysis. (B) GSEA results in the GO analysis. (C–D) The top 20 frequent mutation genes in high- (C) and low-risk (D) patients. (E) Correlations between risk scores and TMB. (F) TMB comparison among risk groups. (G) The survival curves of the high and low TMB groups. (H) OS survival curves in four groups (high-TMB + high-risk score, high-TMB + low-risk score, low-TMB + high-risk score, low-TMB + low-risk score). GSEA, gene set enrichment analysis; OS, overall survival; TMB, tumor mutation burden.

We used GSEA on high-risk and low-risk cohorts, respectively, to deeply investigate the differences in signaling pathways between high-risk and low-risk cohorts. The results of KEGG enrichment analysis of the high-risk group showed that these genes were mainly enriched in cell cycle, ubiquitin-mediated proteolysis, pyrimidine metabolism, pentose phosphate pathway, and oocyte meiosis (Figure 6A). The KEGG enrichment pathway analysis of the low-risk group showed that these genes were mainly involved in the GnRH signaling pathway, Epsilon RI signaling pathway, arachidonic acid metabolism, *α*-linolenic acid metabolism, and vascular smooth muscle contraction (Figure 6A). The GO enrichment analysis results of high-risk groups showed that these genes were mainly enriched in organelle fission, mitotic nuclear division, cytoskeleton-dependent cell division, spindle formation, and mitosis (Figure 6B). These genes in the low-risk group were mainly enriched in histamine transport (Figure 6B). These genes in the high-risk group are mainly involved in the cell cycle and mitotic activities, which may indicate that the tumor phenotype of LUAD patients is more aggressive and prone to rapid proliferation and potential metastasis. In the low-risk group, these genes are mainly involved in metabolic pathways and vascular smooth muscle contraction.

### 3.6. TMB Analysis and Prediction of Immunotherapy Outcomes

TMB plays an important role in the occurrence and development of tumors, and high TMB is associated with tumor genetic instability (genomic instability). Therefore, we analyzed the top 20 genes with increased mutation frequency in LUAD patients. By comparing the mutation rates of 20 genes in high-risk (Figure 6C) and low-risk (Figure 6D), we found that except for zinc finger protein 536 (ZNF536), ankyrin 2 (ANK2), piccolo presynaptic cytomatrix protein (PCLO), CUB and sushi multiple domains 1 (CSMD1), the mutation frequencies of the remaining 16 genes in the high-risk group were significantly higher than those in the low-risk group. Moreover, the mutation frequency of some genes was very different between the low-risk group and the high-risk group. In particular, the TMB of the LUAD-related gene p53 was 51% in the high-risk group and 39% in the low-risk group.

In addition, we performed a correlation analysis between risk score and TMB (Figure 6E), and the correlation analysis results showed that there was a significant positive correlation between risk score and TMB level (*p* < 0.0001). Moreover, the difference in TMB between the high-risk group and the low-risk group (Figure 6F) showed that the TMB in the high-risk group was higher. Comparing the OS results of high-TMB and low-TMB also showed that the survival rate of the high-TMB group was significantly higher than that of the low-TMB group (Figure 6G), which is consistent with the result of significance between risk score and TMB level. We further compared the survival rates of high-TMB + high-risk score, high-TMB + low-risk score, low-TMB + high-risk score, low-TMB + low-risk score, and we found that high-TMB + low LUAD patients with—risk score has the highest survival rate, while low-TMB + high-risk score LUAD patients have the lowest survival rate. High TMB means that there are more mutations in tumor cells that can be recognized by the immune system and trigger an immune response.

### 3.7. The Microenvironment of Tumors and Immune Cell Infiltration Are Associated With Risk Scores

**Figure 7:**
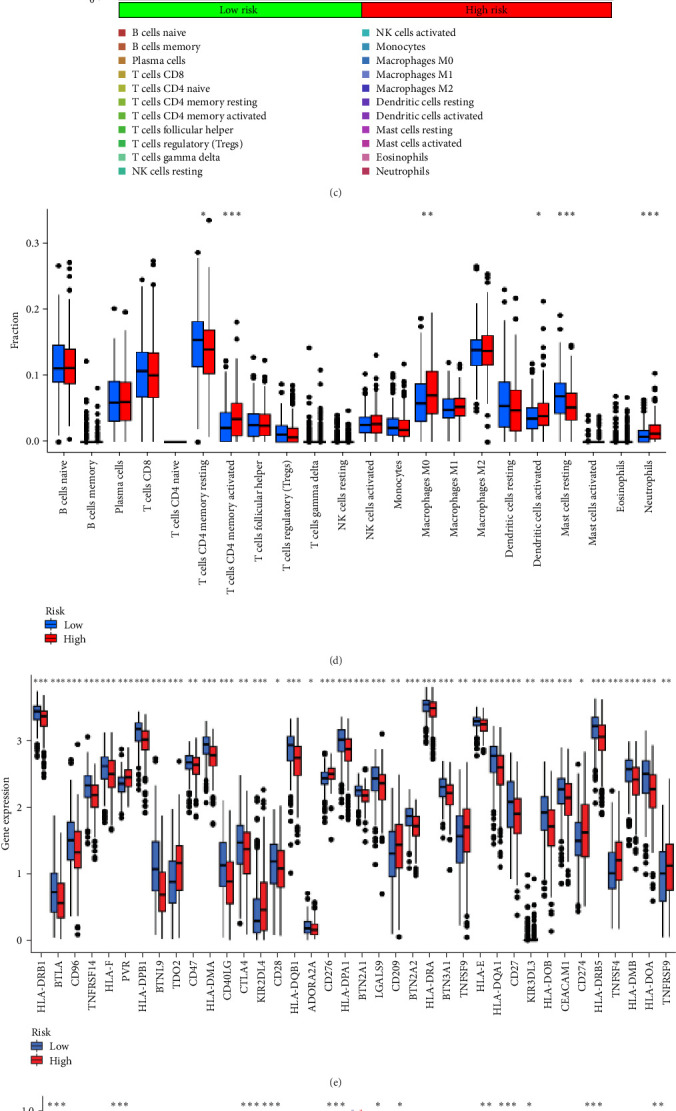
Analysis of immune cell infiltration and related mutations based on the signature. (A) Estimation of tumor purity by immune score. (B) Low-risk and high-risk TIDE scores compared. (C) The infiltration of 22 immune cells by patients at high- and low-risk. (D) Analyzing immune cell infiltration. (E) Identifying immune-related functions. (F) Boxplot showing immune checkpoint expression in high- and low-risk groups. TIDE, tumor immune dysfunction and rejection; TME, tumor microenvironment.

In order to explore the impact of risk score on TME in LUAD patients, we analyzed the infiltration of immune cells in different high-risk and low-risk groups. Compared with the high-risk, the low-risk group showed higher immune, ESTIMATE, and stromal scores (Figure 7A), but only immune and ESTIMATE scores were significantly correlated in the high-risk and low-risk groups (*p* < 0.001). We also used the tumor immune dysfunction and rejection (TIDE) algorithm to evaluate the potential response to immunotherapy in the high-risk group and the low-risk group (Figure 7B). Patients in the low-risk group had significantly higher TIDE scores than those in the high-risk group, indicating that the high-risk group may gain more from immunotherapy. By studying the infiltration of 22 immune cells in high-risk and low-risk patients, we found that all 22 immune cells were infiltrated (Figure 7C). Comparing the differences in the infiltration of 22 immune cells in LUAD patients in the high-risk group and the low-risk group, we found that the infiltration of CD4 memory-activated T cells, macrophages M0, activated dendritic cells, and neutrophils was higher in the risk group, while T Infiltration of CD4 memory cells and mast cells was relatively low (Figure 7D). The study of immune function showed that, except for NK cells, the scores of other cells in the low-risk group were higher than those of the high-risk group (Figure 7E).

Given that cancer immunotherapy critically relies on the activation of immune checkpoints, we explored differences in immune checkpoint gene expression between the two risk groups (Figure 7F). The expression levels of 19 immune checkpoint genes are different between the high-risk group and the low-risk group (*p* < 0.05). The expression levels of BTLA, TNFRSF14, CD200R1, CD40LG, CTLA4, CD48, CD28, ADORA2A, LGALS9, IDO2, and CD44 in the low-risk group are higher than those in the high-risk group, while the expression levels of CD276, TNFSF9, and CD274 were relatively low in the low-risk group. These results indicate that risk score can serve as an important factor that plays a key role in shaping the TME and affecting immune cell function.

### 3.8. Low-Risk Patients May Be More Responsive to Chemotherapy Drugs

**Figure 8:**
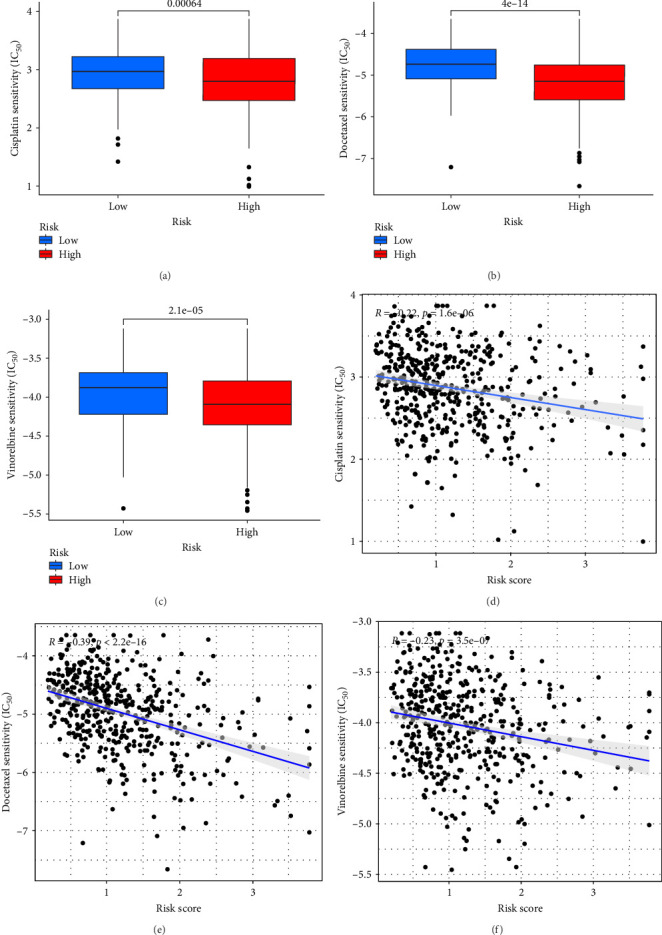
Risk-based drug sensitivity analysis. (A–C) A comparison of drug sensitivity in high-risk and low-risk groups of patients. (D–F) Drug sensitivity and risk score correlation analysis.

This study evaluated the efficacy of three commonly used chemotherapy drugs in the treatment of LUAD patients. It was found that cisplatin (Figure 8A), docetaxel (Figure 8B), and vinorelbine (Figure 8C) were found in LUAD patients in the high-risk group and low-risk group. There was a significant difference in the IC_50_ values (*p* < 0.001), and the IC_50_ values of these three drugs were lower in the high-risk group than in the low-risk group. Correlation analysis between three drug sensitivities and risk scores found that risk scores were negatively correlated with drug sensitivities (Figure 8D–F), so the low-risk group had a more obvious response to chemotherapy drugs.

### 3.9. RT-qPCR Was Used to Confirm the Expression Level of Three LncRNAs in LUAD Cells

**Figure 9:**
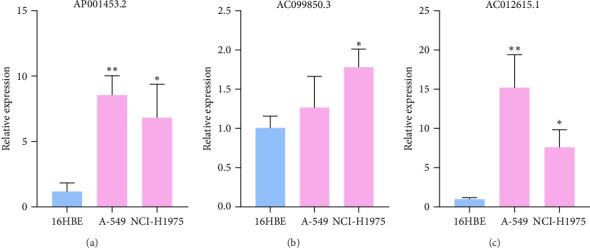
A qRT-PCR analysis validated three differentially expressed lncRNAs. (A) AP001453.2, (B) AC099850.3, (C) AC012615.1. *Note*: *⁣*^*∗*^*p* < 0.05, *⁣*^*∗∗*^*p* < 0.01. LncRNAs long noncoding RNAs, qRT-PCR quantitative real-time PCR.

To verify the accuracy of our prognostic model, we also used qRT-PCR to detect the expression of three lncRNA genes. The results showed that AP001453.2, AC099850.3, and AC012615.1 were significantly upregulated in all LUAD cells compared with 16HBE cells (Figure 9A). This also proved that the expression of three lncRNA genes was correlated with the prognosis of LUAD patients.

## 4. Discussion

### 4.1. LncRNAs and Cancer

LncRNA expression is closely related to various key processes of tumor cells, such as proliferation, invasion, apoptosis, metastasis, and drug resistance [55, 56]. Some studies have shown that specific lncRNAs have different expression patterns in different types of cancer and are closely related to the clinical characteristics, treatment response, and prognosis of tumors [57]. A study has discovered the potential of lncRNAs as valuable biomarkers for cancer diagnosis and prognosis; this study found that various lncRNAs were ectopically expressed in colorectal cancer and that lncRNAs served as prognostic and diagnostic biomarkers for colorectal cancer [58]. Therefore, using lncRNA as biomarkers for early diagnosis, prognosis assessment, and treatment monitoring of cancer has important clinical significance.

LncRNA plays a key regulatory role in the development of cancer. They regulate gene expression through various mechanisms to affect cell growth, differentiation, migration, and apoptosis [20]. LncRNA MALAT1 and HOTAIR are highly expressed in LUAD, promoting tumor cell proliferation, and inhibiting apoptosis by regulating cell cycle proteins and apoptosis-related molecules (such as BCL-2 and BAX) [59]. LncRNA promotes the invasion and metastasis of LUAD cells by regulating EMT. LncRNA LINC01133 is highly expressed in LUAD and can regulate EMT-related factors such as E-cadherin and N-cadherin [60]. LncRNA can directly regulate the expression of target genes by binding to transcription factors or chromatin modification complexes [14]. LncRNA changes the chromatin structure of the CHMP4A gene or directly activates or inhibits its transcription by binding to transcription factors or chromatin modification enzymes. This regulatory method can change the expression level of CHMP4A and affect the signal transduction and metabolic processes in cells. CHMP4A plays an important role in the formation of multivesicular bodies (MVBs) and endocytosis pathways in cells, especially in the process of cell division, proliferation, and migration [61]. In LUAD, abnormal expression of CHMP4A may lead to abnormal transport of intracellular substances and signal transduction to affect the occurrence and development of tumors. A study has found that the isoform pattern of hMENA can affect the formation of tertiary lymphoid structures and immune microenvironment and can also serve as a potential biomarker for predicting the prognosis of NSCLC patients and the therapeutic response to immune checkpoint blockade [62].

### 4.2. Pyroptosis and Cancer

Pyroptosis is a form of programed cell death characterized by inflammation that plays a complex role in cancer [63]. As an important cell death pathway, pyroptosis has also attracted much attention for its application in cancer treatment [64]. It can act as a specific inhibitor or in the tumor suppressor of lytic immune cells. However, it can also trigger the release of inflammatory factors, induce antitumor immunity, and may contribute to tumor progression [65]. Some studies have shown that regulating the pyroptosis pathway can promote tumor cell death and tumor suppression, thereby achieving the purpose of treating cancer [66]. Especially for treatment-resistant tumors, pyroptosis may be an effective therapeutic strategy that can enhance the sensitivity of tumor cells to treatment.

Proptosis plays a key role in the proliferation, invasion, and metastasis of tumors, and its specific effects are often finely regulated by a variety of noncoding RNAs (such as microRNAs, lncRNAs, circular RNAs) and other regulatory molecules [67]. Some microRNAs can affect the activation of pyroptosis by regulating the expression of pyroptosis-related proteins, thereby inhibiting tumor growth and metastasis, such as NLRP3 inflammasomes and GSDMD [68]. In addition, lncRNAs and circRNAs also play an important role in the regulation of pyroptosis signaling pathways [69]. They can affect the pro-inflammatory or pro-immune effects of pyroptosis on tumor cells by competitively binding to microRNAs or regulating the expression of related proteins.

### 4.3. PRLs Model and Other LUAD Prognostic Model

Lung cancer causes more than 1.5 million deaths every year, seriously threatening people's lives and health [70]. There are many types of lung cancer, which brings a lot of trouble to the treatment of lung cancer. A study has found that patients with multiple primary lung cancer (MPLC) have a less inflammatory microenvironment than NSCLC [62]. A multiomics study on 427 SCLC patients found that SCLC subtypes were not only significantly related to patient survival but also closely related to the inflammatory characteristics of tumors [71].

The cancer prognostic model is a tool to predict the progression of a patient's disease and survival based on clinical features, molecular biological data, and individual patient information [72]. The application of the prognostic model is not limited to predicting the patient's survival but can also help clinicians assess the patient's response to different treatments to guide individualized treatment decisions. The lung cancer prognostic model is one of the important tools for precision cancer treatment. A study established a comprehensive gene signature to evaluate the prognosis of LUAD patients through 11 key genes related to cell–cell interactions, tumor development, and T-cell phenotype transformation. Polygenic risk scores are a recent clinical application of whole-genome association studies of lung cancer to predict lung cancer risk [73]. A recent study used data from 4002 lung cancer cases and 20,010 controls to construct and validate a polygenic risk score model based on more than 1.1 million genetic variants [74]. The study found that whole-genome polygenic risk scores are better than previous scoring methods at predicting lung cancer. Moreover, the polygenic risk score further enhances its predictive power after taking into account smoking status. Although such models consider the joint effects of multiple genes, they rely on single or relatively limited biomarkers.

As a cancer prognosis model, the PRLs model combines the biological characteristics of lncRNA and pyroptosis, demonstrating its significant advantages in lung cancer prognosis assessment [75]. PRLs not only provide important information about the tumor immune microenvironment and immune response but can also provide personalized treatment recommendations based on the patient's molecular characteristics. PRLs models are widely used in various cancer models, such as gastric cancer, liver cancer, and renal cancer [76–78]. A study identified 9 PRLs associated with the occurrence and development of gastric cancer from 523 PRLs and constructed a risk prediction model that provides an important reference for the clinical management and individualized treatment of gastric cancer patients [76].

## 5. Conclusion

PRLs genes play a key role in the biological processes of cancer cell proliferation, apoptosis, and metabolic regulation. This study used pRLs to construct a prognostic model for LUAD patients and used the Kaplan–Meier curve and Cox regression analysis to study the prognostic value of pRLs. This prognostic model can not only help clinicians more accurately evaluate the prognosis of LUAD patients but also provide strong support for the formulation of individualized treatment plans. In the future, through in-depth research on the functional mechanisms of these pRL factors, their roles in tumor occurrence, development, and immune microenvironment can be revealed, providing a scientific basis for the targeted treatment and development of new therapies for LUAD.

## Data Availability

This study was conducted based on publicly available databases, including UCSC Xena (https://xena.ucsc.edu/), GeneCards (https://www.genecards.org/), Molecular Signatures Database (MSigDB, http://www.gsea-msigdb.org/gsea/msigdb/).

## Supporting Information

Supporting Information Table S1: The primer sequences for qRT-PCR.

## References

[1] Minna J. D., Roth J. A., Gazdar A. F. (2002). Focus on Lung Cancer. *Cancer Cell*.

[2] Lahiri A., Maji A., Potdar P. D. (2023). Lung Cancer Immunotherapy: Progress, Pitfalls, and Promises. *Molecular Cancer*.

[3] van Meerbeeck J. P., Fennell D. A., De Ruysscher D. K. M. (2011). Small-Cell Lung Cancer. *The Lancet*.

[4] Goldstraw P., Ball D., Jett J. R. (2011). Non-Small-Cell Lung Cancer. *The Lancet*.

[5] Li S., Sun X., Miao S. (2018). hsa_circ_0000729, a Potential Prognostic Biomarker in Lung Adenocarcinoma. *Thoracic Cancer*.

[6] Zhang Y., Wang H., Wang J. (2015). Global Analysis of Chromosome 1 Genes Among Patients With Lung Adenocarcinoma, Squamous Carcinoma, Large-Cell Carcinoma, Small-Cell Carcinoma, or Non-Cancer. *Cancer and Metastasis Reviews*.

[7] Battafarano R. J., Fernandez F. G., Ritter J. (2005). Large Cell Neuroendocrine Carcinoma: An Aggressive Form of Non-Small Cell Lung Cancer. *The Journal of Thoracic and Cardiovascular Surgery*.

[8] Zhu S., Ge T., Hu J., Jiang G., Zhang P. (2021). Prognostic Value of Surgical Intervention in Advanced Lung Adenocarcinoma: A Population-Based Study. *Journal of Thoracic Disease*.

[9] Ruiz-Cordero R., Devine W. P. (2020). Targeted Therapy and Checkpoint Immunotherapy in Lung Cancer. *Surgical Pathology Clinics*.

[10] Sun L., Zhou H., Wu C., Peng Y. (2023). Molecular Markers that Predict Response to Combined Radiotherapy and Immunotherapy in Patients With Lung Adenocarcinoma: A Bioinformatics Analysis. *Translational Cancer Research*.

[11] Alam S. K., Zhang Y., Wang L. (2022). DARPP-32 Promotes ERBB3-Mediated Resistance to Molecular Targeted Therapy in EGFR-Mutated Lung Adenocarcinoma. *Oncogene*.

[12] Yu P., Zhang X., Liu N., Tang L., Peng C., Chen X. (2021). Pyroptosis: Mechanisms and Diseases. *Signal Transduction and Targeted Therapy*.

[13] Zhou C.-B., Fang J.-Y. (2019). The Role of Pyroptosis in Gastrointestinal Cancer and Immune Responses to Intestinal Microbial Infection. *Biochimica et Biophysica Acta (BBA)-Reviews on Cancer*.

[14] Rao Z., Zhu Y., Yang P. (2022). Pyroptosis in Inflammatory Diseases and Cancer. *Theranostics*.

[15] Tan Y., Chen Q., Li X. (2021). Pyroptosis: A New Paradigm of Cell Death for Fighting Against Cancer. *Journal of Experimental & Clinical Cancer Research*.

[16] Wang G., Li B., Tian H. (2023). A Metal-Phenolic Nanocoordinator Launches Radiotherapeutic Cancer Pyroptosis Through an Epigenetic Mechanism. *Advanced Functional Materials*.

[17] Zhang W., Liu Z., Zhu J. (2023). Bioorthogonal Disruption of Pyroptosis Checkpoint for High-Efficiency Pyroptosis Cancer Therapy. *Journal of the American Chemical Society*.

[18] Tsuchiya K. (2021). Switching From Apoptosis to Pyroptosis: Gasdermin-Elicited Inflammation and Antitumor Immunity. *International Journal of Molecular Sciences*.

[19] Hou J., Hsu J.-M., Hung M.-C. (2021). Molecular Mechanisms and Functions of Pyroptosis in Inflammation and Antitumor Immunity. *Molecular Cell*.

[20] Quinn J. J., Chang H. Y. (2016). Unique Features of Long Non-Coding RNA Biogenesis and Function. *Nature Reviews Genetics*.

[21] Dykes I. M., Emanueli C. (2017). Transcriptional and Post-Transcriptional Gene Regulation by Long Non-Coding RNA. *Genomics, Proteomics and Bioinformatics*.

[22] Srijyothi L., Ponne S., Prathama T., Ashok C., Baluchamy S. (2018). Roles of Non-Coding RNAs in Transcriptional Regulation. *Transcriptional and Post-Transcriptional Regulation*.

[23] Statello L., Guo C.-J., Chen L.-L., Huarte M. (2021). Gene Regulation by Long Non-Coding RNAs and Its Biological Functions. *Nature Reviews Molecular Cell Biology*.

[24] Hauptman N., Glavač D. (2013). Long Non-Coding RNA in Cancer. *International Journal of Molecular Sciences*.

[25] Tang L., Wei D., Xu X. (2021). Long Non-Coding RNA MIR200CHG Promotes Breast Cancer Proliferation, Invasion, and Drug Resistance by Interacting With and Stabilizing YB-1. *NPJ Breast Cancer*.

[26] Deng H., Zhang J., Shi J. (2016). Role of Long Non-Coding RNA in Tumor Drug Resistance. *Tumor Biology*.

[27] Jiang X., Wang J., Deng X. (2020). The Role of Microenvironment in Tumor Angiogenesis. *Journal of Experimental & Clinical Cancer Research*.

[28] Guo Y., Xie Y., Luo Y. (2022). The Role of Long Non-Coding RNAs in the Tumor Immune Microenvironment. *Frontiers in Immunology*.

[29] Thapa R., Afzal O., Afzal M. (2024). From LncRNA to Metastasis: The MALAT1-EMT Axis in Cancer Progression. *Pathology-Research and Practice*.

[30] Wang Z., Wu Q., Feng S., Zhao Y., Tao C. (2017). Identification of Four Prognostic LncRNAs for Survival Prediction of Patients With Hepatocellular Carcinoma. *PeerJ*.

[31] Xu F., Huang X., Li Y., Chen Y., Lin L. (2021). m6A-Related lncRNAs Are Potential Biomarkers for Predicting Prognoses and Immune Responses in Patients with LUAD. *Molecular Therapy-Nucleic Acids*.

[32] Zhou M., Hu L., Zhang Z., Wu N., Sun J., Su J. (2018). Recurrence-Associated Long Non-Coding RNA Signature for Determining the Risk of Recurrence in Patients with Colon Cancer. *Molecular Therapy—Nucleic Acids*.

[33] Lonsdale J., Thomas J., Salvatore M. (2013). The Genotype-Tissue Expression (GTEx) Project. *Nature Genetics*.

[34] Tomczak K., Czerwińska P., Wiznerowicz M. (2015). Review the Cancer Genome Atlas (TCGA): An Immeasurable Source of Knowledge. *Współczesna Onkologia*.

[35] Goldman M., Craft B., Hastie M. (2018). The UCSC Xena Platform for Public and Private Cancer Genomics Data Visualization and Interpretation. *Biorxiv*.

[36] Safran M., Dalah I., Alexander J. (2010). GeneCards Version 3: The Human Gene Integrator. *Database*.

[37] Liberzon A., Subramanian A., Pinchback R., Thorvaldsdóttir H., Tamayo P., Mesirov J. P. (2011). Molecular Signatures Database (MSigDB) 3.0. *Bioinformatics*.

[38] Fu L., Niu B., Zhu Z., Wu S., Li W. (2012). CD-HIT: Accelerated for Clustering the Next-Generation Sequencing Data. *Bioinformatics*.

[39] Yang Y., Chen H.-L., Wu S. F. (2024). Bao W: Chmp4b and Vsp4A Reverse Gsdmd-Mediated Pyroptosis by Cell Membrane Remodeling in Endometrial Carcinoma. *Biochimica et Biophysica Acta (BBA)-General Subjects*.

[40] Moll U. M., Slade N. (2004). p63 and p73: Roles in Development and Tumor Formation. *Molecular Cancer Research*.

[41] Lin W., Chen Y., Wu B., Chen Y., Li Z. (2021). Identification of the Pyroptosis-Related Prognostic Gene Signature and the Associated Regulation Axis in Lung Adenocarcinoma. *Cell Death Discovery*.

[42] Dong Z., Bian L., Wang M., Wang L., Wang Y. (2021). Identification of a Pyroptosis-Related Gene Signature for Prediction of Overall Survival in Lung Adenocarcinoma. *Journal of Oncology*.

[43] Ritchie M. E., Phipson B., Wu D. (2015). Limma Powers Differential Expression Analyses for RNA-Sequencing and Microarray Studies. *Nucleic Acids Research*.

[44] Smoot M. E., Ono K., Ruscheinski J., Wang P.-L., Ideker T. (2011). Cytoscape 2.8: New Features for Data Integration and Network Visualization. *Bioinformatics*.

[45] Martinussen T. (2022). Causality and the Cox Regression Model. *Annual Review of Statistics and Its Application*.

[46] Kukreja S. L., Löfberg J., Brenner M. J. (2006). A Least Absolute Shrinkage and Selection Operator (LASSO) for Nonlinear System Identification. *IFAC Proceedings Volumes*.

[47] Iasonos A., Schrag D., Raj G. V., Panageas K. S. (2008). How to Build and Interpret a Nomogram for Cancer Prognosis. *Journal of Clinical Oncology*.

[48] Harrell F. E., Harrell M. F. E. (2017). *Package ‘Rms’*.

[49] Abdi H., Williams L. J. (2010). Principal Component Analysis. *WIREs Computational Statistics*.

[50] Subramanian A., Tamayo P., Mootha V. K. (2005). Gene Set Enrichment Analysis: A Knowledge-Based Approach for Interpreting Genome-Wide Expression Profiles. *Proceedings of the National Academy of Sciences of the United States of America*.

[51] Young M. D., Wakefield M. J., Smyth G. K. (2010). Oshlack A: Gene Ontology Analysis for RNA-Seq: Accounting for Selection Bias. *Genome Biology*.

[52] Kanehisa M., Furumichi M., Tanabe M., Sato Y., Morishima K. (2017). KEGG: New Perspectives on Genomes, Pathways, Diseases and Drugs. *Nucleic Acids Research*.

[53] Kassambara A., Kassambara M. A. (2020). Package *01*8;ggpubr. *R Package Version*.

[54] Wilkinson L. (2011). *ggplot2:Elegant Graphics for Data Analysis by WICKHAM, H*.

[55] Li J., Meng H., Bai Y., Wang K. (2016). Regulation of lncRNA and Its Role in Cancer Metastasis. *Oncology Research*.

[56] Heery R., Finn S. P., Cuffe S., Gray S. G. (2017). Long Non-Coding RNAs: Key Regulators of Epithelial-Mesenchymal Transition, Tumour Drug Resistance and Cancer Stem Cells. *Cancers*.

[57] Ghafouri-Fard S., Shoorei H., Dashti S., Branicki W., Taheri M. (2020). Expression Profile of lncRNAs and miRNAs in Esophageal Cancer: Implications in Diagnosis, Prognosis, and Therapeutic Response. *Journal of Cellular Physiology*.

[58] Dastmalchi N., Safaralizadeh R., Nargesi M. M. (2020). LncRNAs: Potential Novel Prognostic and Diagnostic Biomarkers in Colorectal Cancer. *Current Medicinal Chemistry*.

[59] Poulet C., Njock M.-S., Moermans C. (2020). Exosomal Long Non-Coding RNAs in Lung Diseases. *International Journal of Molecular Sciences*.

[60] Tang P. C.-T., Zhang Y.-Y., Li J. S.-F. (2022). LncRNA-Dependent Mechanisms of Transforming Growth Factor-*β*: From Tissue Fibrosis to Cancer Progression. *Non-Coding RNA*.

[61] Wang Z.-N., Ma H.-M., Z-y Chen (2022). Pan-Cancer Prognostic, Immunity, Stemness, and Anticancer Drug Sensitivity Characterization of Pyroptosis Related Genes in Human Cancers.

[62] Di Modugno F., Di Carlo A., Spada S. (2024). Tumoral and Stromal hMENA Isoforms Impact Tertiary Lymphoid Structure Localization in Lung Cancer and Predict Immune Checkpoint Blockade Response in Patients With Cancer. *EBioMedicine*.

[63] Chen B., Yan Y., Yang Y. (2022). A Pyroptosis Nanotuner for Cancer Therapy. *Nature Nanotechnology*.

[64] Huang Y., Wang J.-W., Huang J. (2022). Pyroptosis, a Target for Cancer Treatment?. *Apoptosis*.

[65] Kim R., Emi M., Tanabe K. (2014). Cancer Cell Immune Escape and Tumor Progression by Exploitation of Anti-Inflammatory and Pro-Inflammatory Responses. *Cancer Biology & Therapy*.

[66] Zamarron B. F., Chen W. J. (2011). Dual Roles of Immune Cells and Their Factors in Cancer Development and Progression. *International Journal of Biological Sciences*.

[67] Chen S., Navickas A., Goodarzi H. (2024). Translational Adaptation in Breast Cancer Metastasis and Emerging Therapeutic Opportunities. *Trends in Pharmacological Sciences*.

[68] Vande Walle L., Lamkanfi M. (2024). Drugging the NLRP3 Inflammasome: From Signalling Mechanisms to Therapeutic Targets. *Nature Reviews Drug Discovery*.

[69] Gao L., Jiang Z., Han Y., Li Y., Yang X. (2022). Regulation of Pyroptosis by ncRNA: A Novel Research Direction. *Frontiers in Cell and Developmental Biology*.

[70] Leiter A., Veluswamy R. R., Wisnivesky J. P. (2023). The Global Burden of Lung Cancer: Current Status and Future Trends. *Nature Reviews Clinical Oncology*.

[71] Park S., Hong T. H., Hwang S. (2024). Comprehensive Analysis of Transcription Factor-Based Molecular Subtypes and Their Correlation to Clinical Outcomes in Small-Cell Lung Cancer. *EBioMedicine*.

[72] Mallett S., Royston P., Waters R., Dutton S., Altman D. G. (2010). Reporting Performance of Prognostic Models in Cancer: A Review. *BMC Medicine*.

[73] Xu J., Zhang Y., Li M. (2024). A Single-Cell Characterised Signature Integrating Heterogeneity and Microenvironment of Lung Adenocarcinoma for Prognostic Stratification. *EBioMedicine*.

[74] Boumtje V., Manikpurage H. D., Li Z. (2024). Polygenic Inheritance and Its Interplay With Smoking History in Predicting Lung Cancer Diagnosis: A French-Canadian Case-Control Cohort. *EBioMedicine*.

[75] Altman D. G., Vergouwe Y., Royston P., Moons K. G. M. (2009). Prognosis and Prognostic Research: Validating a Prognostic Model. *BMJ*.

[76] Wang Y., Li D., Xun J., Wu Y., Wang H.-L. (2024). Construction of Prognostic Markers for Gastric Cancer and Comprehensive Analysis of Pyroptosis-Related Long Non-Coding RNAs. *World Journal of Gastrointestinal Surgery*.

[77] Qu G., Wang D., Xu W., Guo W. (2022). Comprehensive Analysis of the Correlation Between Pyroptosis-Related LncRNAs and Tumor Microenvironment, Prognosis, and Immune Infiltration in Hepatocellular Carcinoma. *Frontiers in Genetics*.

[78] Zhou X., Yao L., Zhou X. (2022). Pyroptosis-Related lncRNA Prognostic Model for Renal Cancer Contributes to Immunodiagnosis and Immunotherapy. *Frontiers in Oncology*.

