# Supplementary material for: Development of a Novel Prognostic Model for Lung Adenocarcinoma Utilizing Pyroptosis-Associated LncRNAs
Source: Anal Cell Pathol (Amst). 2025 Jan 13;2025:4488139. doi: 10.1155/ancp/4488139 (PMC11745560; doi:10.1155/ancp/4488139)
Supplement: Supporting Information — Table S1: The primer sequences for qRT-PCR. [file 4488139.f1.docx]

Table S1.The primer sequences for qRT-PCR

| Primer | Sequence(5-3） |
| --- | --- |
| GAPDH-F | GAC CTG ACC TGC CGT CTA |
| GAPDH-R | AGG AGT GGG TGT CGC TGT |
| AP001453.2-F | CCCCCACAACATCCAAAGAG |
| AP001453.2-R | TTCCTCAGAAGGCTTGTGCC |
| AC099850.3-F | CCTTCCACCTGATGGCCTTT |
| AC099850.3-R | TTAAGAACCCCACGGAGCAC |
| AC012615.1-F | TTGGGTGGATGGTGGTGGTTT |
| AC012615.1-R | GGGTCAGCCGAGAAATGGTATTCA |
